# Peculiarity of the Structure and Luminescence of Glasses in La_2_S_3_-Ga_2_S_3_-GeS_2_:Pr^3+^ System

**DOI:** 10.3390/ma16227094

**Published:** 2023-11-09

**Authors:** Andrey Tverjanovich, Egor Smirnov

**Affiliations:** Institute of Chemistry, St. Petersburg State University, 198504 St. Petersburg, Russia; gorsmirnov@mail.ru

**Keywords:** chalcogenide glasses, luminescent materials, IR materials

## Abstract

The effect of modifying the composition of a glass matrix based on the Ga_2_S_3_-GeS_2_:Pr^3+^ system due to the addition of La_2_S_3_ on the structure and the optical and luminescent properties of these glasses has been studied. It has been shown that the addition of La_2_S_3_ leads to changes in the nearest structural environment of Ga, Ge, and S and increases the degree of ionicity of the bonds of the Pr^3+^ ion. Despite the existence of a large glass formation region in the Ga_2_S_3_-GeS_2_-La_2_S_3_ system and the structural and chemical similarity of La and Pr, La_2_S_3_ does not promote a more uniform distribution of Pr^3+^ ions in the glass matrix, and thus does not reduce the concentration quenching of the luminescence of Pr^3+^ ions. However, the addition of La_2_S_3_ increases the probability of emission of Pr^3+^ ions and decreases the radiative lifetime. Additionally, it was shown that, when studying the structure and luminescent properties of glasses with La, it is necessary to take into account a significant concentration of rare earth traces (Pr and Nd).

## 1. Introduction

Chalcogenide glasses (ChGs) possess a number of properties that make them attractive as a matrix for rare earth ions (REIs) to create luminescent materials for the near- and mid-IR spectral region. These properties include low phonon energy, high refractive index, and transparency across a wide spectral range. Furthermore, by adjusting the composition, glasses can be selected that exhibit optimal values of glass transition temperature and crystallization stability. Recently, interest in ChGs doped with REIs has resurged due to the development of active optical fibers based on them for the mid-IR region of the spectrum [1]. The demand for such fibers stems from the possibility of overlapping the region of characteristic vibrations of the organic groups, which makes it possible to create portable gas sensors based on such fibers as well as to use them in biological and medical diagnostics [2,3,4].

It is believed that RE elements are relatively well soluble in ChGs [5,6]. However, this pertains solely to obtaining a material in a glassy state, and not to the degree of uniformity in the distribution of REIs within the glass matrix. To prevent nonradiative relaxation of an excited REI due to energy transfer to another REI, the latter ion must be located beyond the second coordination sphere. With a uniform distribution of REIs in the glass matrix, this condition is satisfied by an REI concentration of ≤6 at.% [7]. At the same time, in reality, concentration quenching transpires at concentrations less than 1 at.% REI, indicating the uneven distribution of REIs in the glass matrix and the formation of regions enriched with REIs.

To enhance the solubility of REIs in chalcogenide glasses, several strategies were employed. First of all, glasses containing Ga_2_S_3_ were used as a matrix. Ga in chalcogenide glasses forms GaS_4_^−^ tetrahedral complex structural units, which compensate for the REI charge and contribute to its more uniform distribution [8,9]. Second, La_2_S_3_ forms a large region of glass formation with Ga_2_S_3_ and GeS_2_ [5]. Along the La_2_S_3_-GeS_2_ quasi-binary section, up to 50 mol.% La_2_S_3_ can be incorporated, and along the La_2_S_3_-Ga_2_S_3_ section, the range spans from 25 to 50 mol.% La_2_S_3_. Based on this premise, it was a priori believed that significant amounts of other RE elements should homogeneously dissolve in glasses with a high content of La_2_S_3_ because they should isomorphically replace the positions of the lanthanum atoms [10,11]. From this point of view, Pr is the most suitable RE element because it is the nearest element to La in the lanthanide series and its ionic radius should be closest to the ionic radius of La at the same coordination number. With an increase in the atomic number of an RE element, the ionic radius should decrease due to the compression effect [12].

At the same time, during the investigation of the luminescent properties of REIs in chalcogenide glasses containing lanthanum, the most effective concentrations of RE elements—excluding lanthanum—did not exceed, for example, 0.08 at.% Dy [13], 1.2 at.% Er [14], and 0.04 at.% Nd [15].

In this regard, the following question arises: Do ions of other lanthanides in chalcogenide glass containing La really occupy structural positions equivalent to La? Such an arrangement would contribute to their dissolution in the glass matrix and consequently lead to a reduction in the concentration quenching of their luminescence.

To solve this issue, a Ga_2_S_3_-GeS_2_ quasi-binary system was chosen as the basic glass-forming system. Pr was chosen as the RE element. This choice was due to its structural and chemical similarity to La. Furthermore, the Pr^3+^ ion has a number of practical advantages. In chalcogenide glass doped with Pr^3+^ ions, a broad luminescence band is observed in the region from 2000 to 2700 nm [16], which overlaps the fourth transparency window of biological tissues (2100–2300 nm). This spectral window is well suited for studying collagen-containing tissues [17,18]. Also, broadband luminescence in the mid-IR region of the spectrum from 3.5 to 5.2 μm makes it possible to create gas optical sensors for CO_2_, CO, and N_2_O [19,20].

The objective of this work was to study the effect of modifying the composition of the glass matrix based on the Ga_2_S_3_-GeS_2_ quasi-binary system by adding La_2_S_3_ on the concentration quenching of the luminescence of Pr^3+^ ions, as well as to reveal the features of the luminescence and structure of these glasses. The relatively high refractive index, transparency in the IR range, and small radiative lifetime REIs in these glasses allow us to consider them as optically active materials for fiber optics in the near- and mid-IR range.

As a result of the study, the influence of La_2_S_3_ on the structure and properties of glasses of the Ga_2_S_3_-GeS_2_ system was studied. It is shown that the introduction of La_2_S_3_ leads to some changes in the nearest structural environment of Ga, Ge, and S. Furthermore, the addition of La_2_S_3_ increases the degree of ionicity of the bond between the Pr^3+^ ion and sulfur. In terms of its influence on the luminescence properties of Pr^3+^ ions, La_2_S_3_ does not help to reduce the concentration quenching of the luminescence of Pr^3+^ ions in these glasses. Nevertheless, it increases the probability of emission and reduces the radiative lifetime. Apparently, the positions occupied by the La^3+^ and Pr^3+^ ions in the glass structure are not equivalent. Moreover, the study highlighted the importance of considering the significant concentration of rare earth impurity elements (Pr and Nd) when studying the structure and luminescent properties of glasses with La.

## 2. Materials and Methods

### 2.1. Materials

The compositions for synthesis and research were chosen as follows. First, the fraction of La_2_S_3_ was varied while keeping the ratio of Ga_2_S_3_ and GeS_2_ constant. Second, the ratio of Ga_2_S_3_ and GeS_2_ was varied at a constant fraction of La_2_S_3_. This choice of compositions makes it possible to trace the influence of each of the components on the properties under investigation. The chemical composition of the synthesized glasses used for introducing Pr is given in Table 1.

Synthesis was carried out by fusing pure components (Sigma-Aldrich, Saint Louis, MO, USA) in quartz ampoules, which were evacuated to high vacuum and sealed. The furnace constantly wobbled during the synthesis. Heating was conducted in several stages due to the high pressure of the sulfur vapor at the initial stages of synthesis. To prevent interaction of La with the walls of the quartz ampoule at high temperatures, the inside of the ampoule was coated with glass graphite (see Supplementary Materials). The maximum synthesis temperature was 1050 °C. All glasses were obtained by quenching the ampoules with a melt in an air environment. The prepared glassy matrixes were used as a host for the further preparation of glasses containing various concentrations of Pr^3+^ ions. Synthesis of glasses containing Pr was carried out using a similar procedure, but in one stage.

The composition of the synthesized samples was checked using EDX analysis (EDX 800P, Shimadzu, Kyoto, Japan).

### 2.2. Methods

To measure the properties of the samples, plane-parallel plates with a thickness of about 2 mm were obtained by grinding and polishing the synthesized glasses. The exact thickness values of these samples were measured using a high-precision digital snap gage.

The density of the obtained glasses was measured with hydrostatic weighing in toluene. The refractive index n was determined by observing the change in the position of the focal plane of the lens when a plane-parallel sample was placed between the focus and the lens. In such cases, the refractive index can be calculated using the following equation: n = (1 − Δx/d)^−1^, where d is the thickness of the sample and Δx is the difference in the positions of the focal plane without and with the sample. These measurements were carried out at a wavelength of 1.2 μm using an infrared IR microscope (GOI, St. Petersburg, Russia).

The absence of crystalline inclusions in the samples was controlled with X-ray diffraction using a D2 Phaser diffractometer (Bruker, Billerica, MA, USA) with the following parameters: CuKα_1+2_ X-ray tube radiation, sample rotation speed 20 rpm, diffraction angle interval 2 theta = 7–60°, scanning step of 0.02°.

Measurements of the optical absorption spectra within the spectral range of 0.5 to 3.2 µm were carried out using a UV-3600 spectrometer (Shimadzu, Kyoto, Japan), while spectra spanning the range from 2.5 to 25 µm were taken on a Tensor 27 spectrophotometer (Bruker, USA). The instrument ranges overlapped to ensure comprehensive data coverage.

The Raman spectra of glassy samples were measured using a Senterra Raman spectrometer (Bruker, USA) coupled with an Olympus BX-52 optical microscope (Olympus Optical, Tokyo, Japan). These measurements were taken at room temperature. A laser with a wavelength of 785 nm served as the excitation source. The laser power was 1 mW to prevent any potential sample heating.

Luminescence spectra were measured using a Fluorolog-3 spectrofluorometer (Horiba Jobin Yvon, Osaka, Japan). The pump laser beam was directed at the sample surface at an angle of approximately 90 degrees. The signal-collecting optical fiber was positioned at an angle of approximately 45 degrees to this surface at a distance of approximately 2 cm from the surface. In this geometry, the reflected beam of the pump laser did not enter into the light guide. To determine the most effective excitation wavelength, the luminescence excitation spectrum was measured at a wavelength of 1040 nm. Subsequently, the determined value of the pump wavelength (607 nm) was used to excite luminescence.

The calculation of radiative lifetime, transition probability, and branching coefficients in accordance with the Judd–Ofelt theory from the optical absorption spectra was carried out using the methodology outlined in [21]. The matrix elements for these calculations were sourced from [22].

## 3. Results and Discussion

All the samples, including the matrixes themselves and the samples containing praseodymium ions, were obtained in the glassy state by cooling the ampoules with the melt in air. The absence of crystalline inclusions in the glass samples was controlled with X-ray diffraction (Figure S1A in Supplementary Materials). An example of a photo image of the synthesized glasses is shown in Figure S1B.

### 3.1. Optical Absorption Spectroscopy

**Figure 1:**
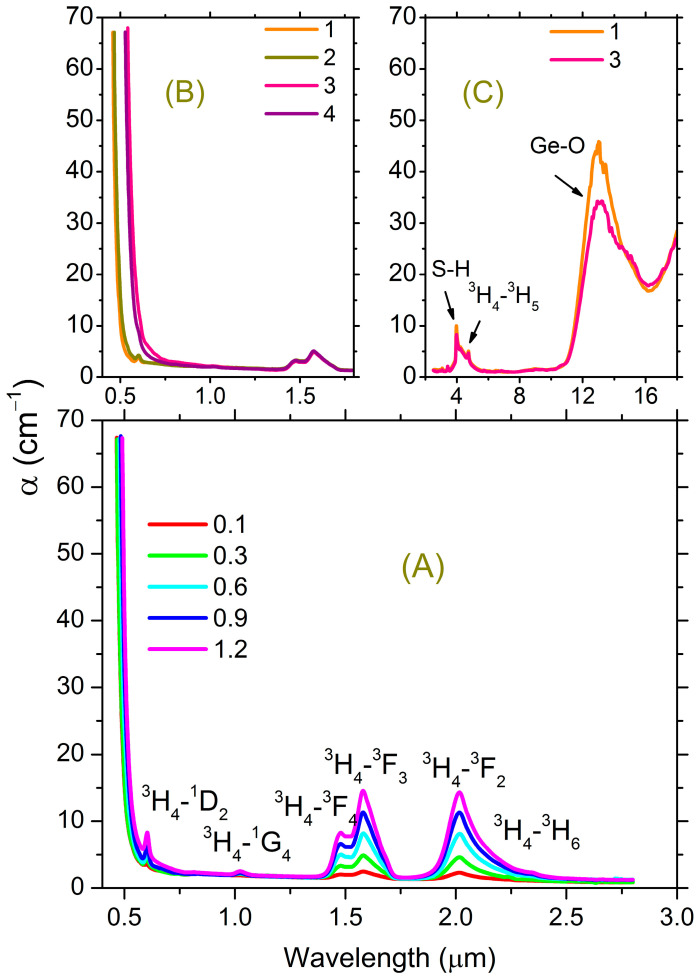
(**A**) Absorption spectra of glass composition 1 with Pr content from 0.1 to 1.2 at.%. (**B**) Absorption spectra of glasses of the studied compositions containing 0.3 at.% Pr. (**C**) Absorption spectra of glasses of compositions 1 and 3 containing 1.2 at.% Pr in the IR range.

The introduction of Pr into the studied glasses leads to an insignificant, monotonic shift of the fundamental absorption edge toward the longer wavelength region of the spectrum. Figure 1A shows the absorption spectra of glass composition 1 with varying Pr content.

The edge shifts by approximately 2.3 nm with an increase in concentration of 0.1 at.%. The band used to excite the luminescence is located in the region of the weak absorption tail. Similarly, the introduction of La_2_S_3_ into the glasses while maintaining a constant concentration of Pr also leads to a shift of the absorption edge toward the longer-wavelength region of the spectrum (Figure 1B). Therefore, the introduction of 10 mol.% LaS_1.5_ (~3.5 at.% La) leads to a shift of the short-wavelength absorption edge by ~85 nm, corresponding on average to ~2.4 nm per 0.1 at.% increase in La concentration. Thus, the degree of influence of the Pr and La additives on the spectral position of the fundamental absorption edge of the studied glasses is approximately the same.

This bias has two components. These are the metal-to-metal bonds discussed in the Raman section and the difference in band gap values of the corresponding sulfides. For La_2_S_3_ in the thin film state, Eg = 2.5 eV [23]. The band gap is much larger for GeS_2_ and Ga_2_S_3_. For Ga_2_S_3_, the band gap varies from 2.74 to 3.30 eV according to various references [24,25,26]. In the case of GeS_2_, Eg ranges from 3.1 to 3.25 eV [27,28].

In the IR region of the spectrum, the introduction of La_2_S_3_ into glasses has practically no effect on the absorption spectrum of the glass (Figure 1C). The position of the phonon absorption edge in the spectrum is determined by the presence of Ge-O impurity bonds. Absorption on impurity S-H groups is also observed, which partially overlaps the broadband absorption due to the ^3^H_4_-^3^H_5_ transition of the Pr^3+^ ion (Figure 1C).

**Figure 2:**
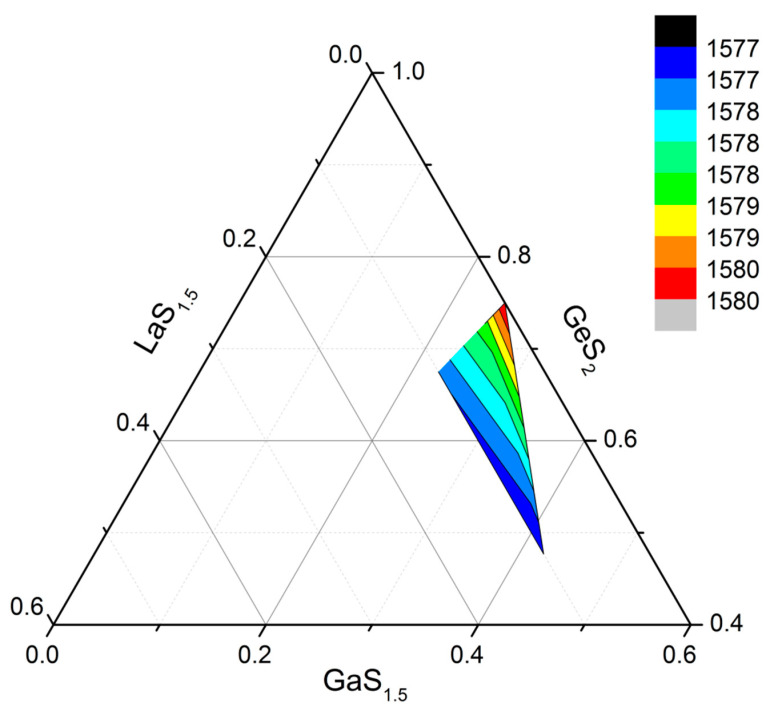
Spectral position of the absorption band of the Pr^3+^ ion due to the transition ^3^H_4_-^3^F_3_, depending on the composition of the glass.

The spectral position of the absorption bands for REIs depends, among other factors, on the interaction of the absorbing ion with the surrounding atoms, namely, on the strength of the local electric field on the lanthanide ion, on the degree of ionicity of the realized bonds, and on the coordination number. Thus, in chalcogenide glasses containing REIs, a spectral shift of the REI absorption bands is observed with a change in the degree of bond ionicity upon replacement of a chalcogen in a series of group 6 elements [7]. In this context, it should be taken into account that the coordination number for REIs is determined by the ratio of the cation and anion radii—Pr and S, in this case. Consequently, the coordination number of Pr should not depend on the composition of the glassy matrix in the studied systems. The shift in the spectral position of the absorption bands should thus be attributed to changes in the degree of ionicity of the bonds realized by Pr^3+^ ions. If we construct the spectral position of the Pr^3+^ absorption band in dependence on the composition of the glassy matrix in the studied glasses (Figure 2), then regularities are visible.

Upon adding La_2_S_3_ to the glasses, the degree of ionicity of the bonds of the Pr^3+^ ion increases. The ratio of Ge to Ga has a weak influence on the spectral position of the absorption band of the Pr^3+^ ion, and consequently, on the degree of ionicity of the bond between the Pr^3+^ ion and sulfur. An increase in the degree of ionicity of praseodymium–sulfur bonds should correlate with an increase in the electronegativity of metal atoms in the second coordination sphere. Pauling electronegativity is as follows: Ge = 2.0, Ga = 1.8, La = 1.1, and Pr = 1.1. Apparently, the mechanism of influence of La is different.

### 3.2. The Structure of the Studied Glasses according to Raman Spectroscopy Data

#### 3.2.1. Initial Glasses of the Ga_2_S_3_-GeS_2_ System and the Influence of the La_2_S_3_ Addition

**Figure 3:**
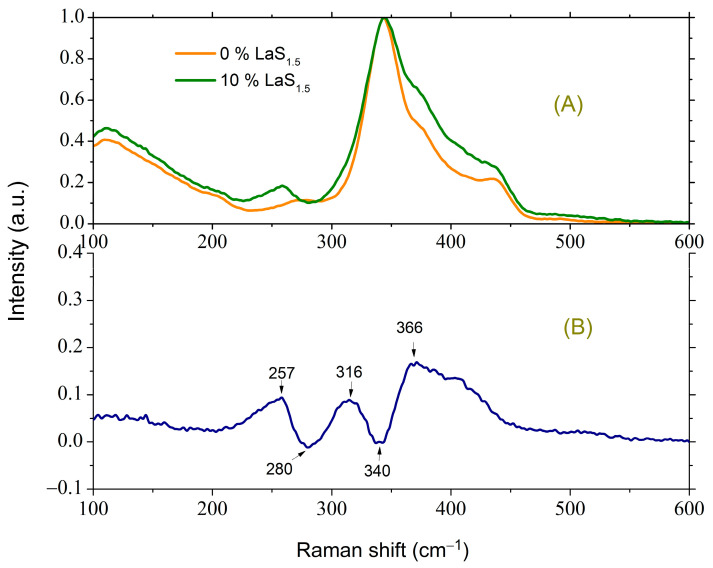
(**A**) Raman spectra of glasses of composition 1 and 3. (**B**) The difference between the normalized Raman spectra of glasses of composition 3 and 1.

The Raman spectra of the initial glass of composition 1 and after the introduction of 10 mol.% LaS_1.5_ (composition 3) are shown in Figure 3A.

For enhanced clarity, the changes occurring in the spectrum of glass with the introduction of 10 mol.% LaS_1.5_ are presented as the difference spectrum in Figure 3B. This difference spectrum is obtained as the spectrum of the glass of composition 3 normalized to the most intense peak (344 cm^−1^) minus the normalized spectrum of the glass of composition 1. The peak at 342–344 cm^−1^ corresponds to the fully symmetric vibration of the GeS_4/2_ tetrahedron [29,30,31].

For glass containing 10 mol.% LaS_1.5_, excess scattering is observed in the regions of 257, 316, and 366 cm^−1^. In the latter case, a broad asymmetric peak is present. The peak at 257 cm^−1^ corresponds to vibrations of the S_3/2_Ge-GeS_3/2_ bonds [32,33], while in the initial glass without La_2_S_3_, the peak due to metal–metal bonds is located at 272 cm^−1^, indicative of Ga-Ga vibrations in a similar structural unit [8,31,34]. Hence, it can be inferred that the introduction of La_2_S_3_ induces a redistribution of S between tetrahedra based on Ge and Ga, favoring the latter. This conclusion aligns with the increase in scattering observed at 316 cm^−1^ because the peak in the region of 320 cm^−1^ can be attributed to vibrations of GaS_4/2_^−^ tetrahedra [8,34,35] or vibrations of three tetrahedra connected by a common vertex [31]. As for the broad asymmetric peak from 350 to 450 cm^−1^, the calculation shows that this range can contain one of the modes due to vibrations of three Ga-based tetrahedra connected by a common vertex [31]. These results indicate both an increase in the fraction of GaS_4_ tetrahedra and an increase in the fraction of three-coordinated sulfur. Moreover, based on references [9,36,37], the band at 375 cm^−1^ arises from vibrations of GeS_4_ and/or GaS_4_ tetrahedra containing at least one non-bridging sulfur. With the addition of La_2_S_3_, the features characteristic of GeS_2_ in the spectrum in the region of 370 and 433 cm^−1^, corresponding to the vibrations of GeS_4/2_ tetrahedra connected by edges [30,31], become less pronounced (Figure 3A), suggesting a decrease in the proportion of such structural units.

**Figure 4:**
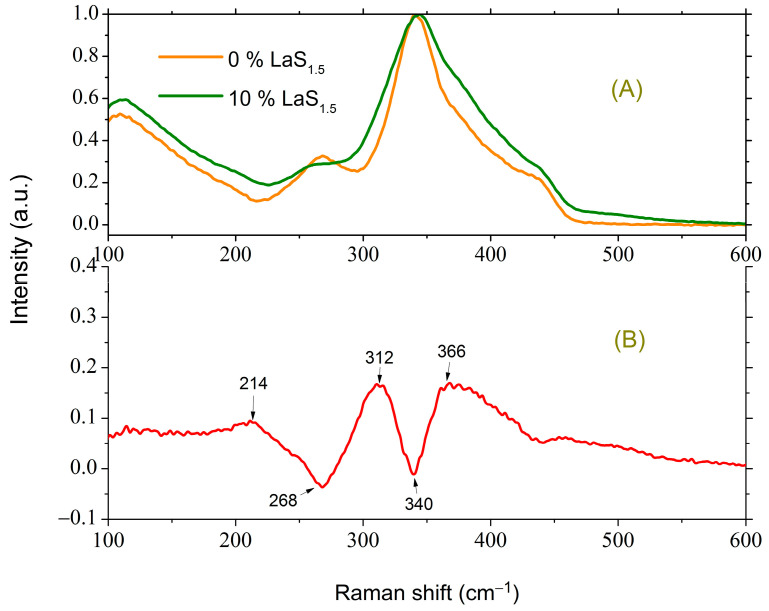
(**A**) The normalized Raman spectra of glasses of compositions 4 and 5. (**B**) The difference between the normalized Raman spectra of glasses of compositions 5 and 4.

When La_2_S_3_ is introduced into glass containing a larger proportion of Ga_2_S_3_ (composition 4), the changes in the Raman spectrum that occur are somewhat different. Figure 4A displays the Raman spectra of the initial glass of composition 4 and the glass after introducing 10 mol.% LaS_1.5_ (composition 5).

Figure 4B presents the difference spectrum, which is obtained by normalizing the spectrum of glass of composition 5 to the most intense peak (342 cm^−1^) and then subtracting the normalized spectrum of glass of composition 4. 

The main difference between this spectrum and the spectrum presented in Figure 3 is observed at shift values less than 280 cm^−1^. In the initial glass (composition 4), the peak associated with metal–metal bonds is in the region of 267 cm^−1^ (Figure 4A). This indicates that if in the cases of composition 1 the metal–metal peak is mainly due to Ga-Ga bonds, as indicated above, then in the cases of composition 4 there is an additional contribution from Ge-Ge bonds and/or Ge-Ga bonds. Upon introducing 10 mol.% LaS_1.5_, the peak shifts to the region of frequencies corresponding to Ge-Ge bond vibrations (261 cm^−1^) as in the case of glass of composition 1. However, in this case, the intensity of this peak not only does not increase, but even decreases, resulting in a dip in the region of 268 cm^−1^ in the difference spectrum. This decrease may be related to the relatively lower content of GeS_2_ in the glass. A decrease in the intensity of the peak due to metal–metal bonds was also observed in [10] upon transition from the glass composition 80GeS_2_-20Ga_2_S_3_ to the composition 80GeS_2_-15Ga_2_S_3_-05La_2_S_3_. A slight increase in scattering around 214 cm^−1^ could be tentatively attributed to the vibrations of the La-S bonds (typically around 220 cm^−1^ [9,38]). In the case of glass 3, the contribution from the La-S bonds seems to overlap with the contribution from the Ge-Ge bonds, as evidenced by the asymmetry of the peak with a maximum at 257 cm^−1^ (see Figure 3).

Summarizing the above, it should be concluded that the introduction of La_2_S_3_ leads to an increase in the relative content of GaS_4_ tetrahedra, a decrease in the proportion of Ga-Ga bonds, an increase in the proportion of three-coordinated and non-bridging sulfur, and a decrease in the proportion of edge-connected GeS_4_ tetrahedra.

#### 3.2.2. Influence of the Introduction of Pr on the Structure of Glasses according to Raman Spectroscopy Data

**Figure 5:**
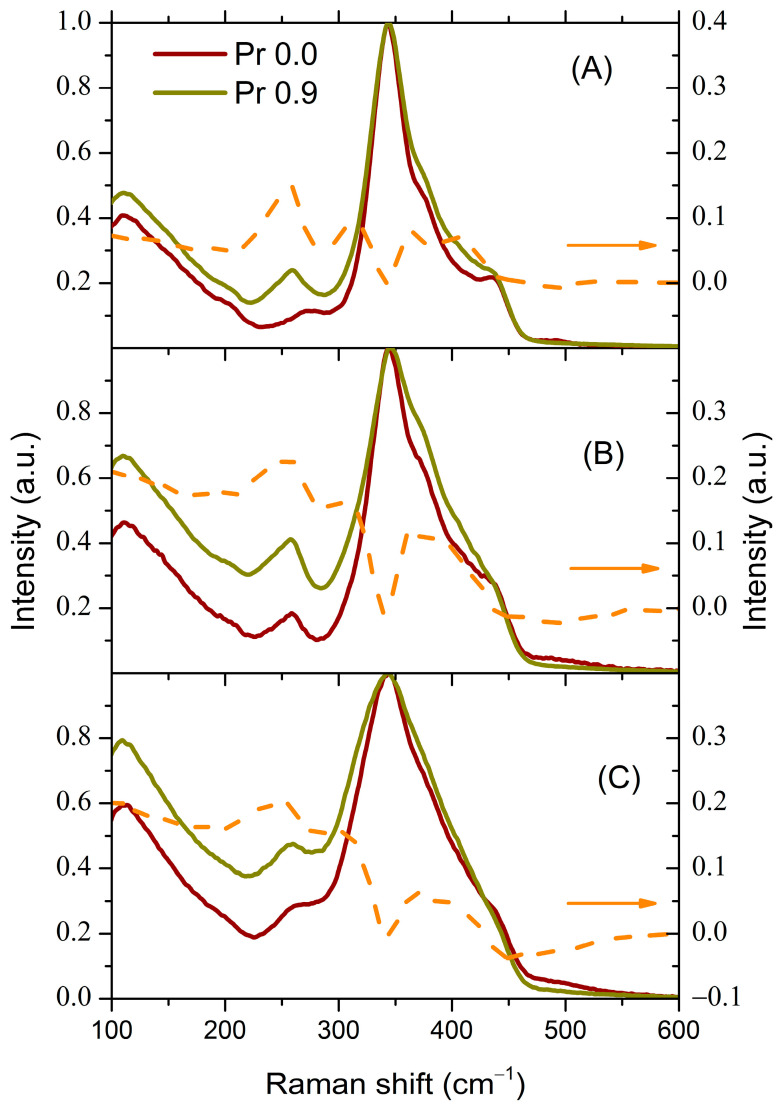
Raman spectra of glasses containing 0.9 at.% Pr. (**A**) Glass of composition 1; (**B**) glass of composition 3; (**C**) glass of composition 5. The dashed orange curve shows the corresponding differences in the Raman spectra of these glasses: the Raman spectrum of the glass containing 0.9 at.% Pr minus the spectrum of the original glass.

Upon introducing Pr into glass of composition 1, the same main changes are observed in the Raman spectrum (Figure 5A) as when La is added (Figure 3A).

Despite the proximity of Pr and La in the lanthanide series, the effect of Pr on the glass structure is much stronger. The impact of introducing less than a percent of Pr is comparable to that of adding 10 mol.% LaS_1.5_. It can be assumed that Pr coordinates Ga-based structural units around itself much more efficiently.

The introduction of Pr into glasses containing 10 mol.% LaS_1.5_ (compositions 3 and 5) does not lead to a significant change in the spectral position of the peak due to metal–metal bonds (Figure 5B,C), but only results in a slight increase in its intensity.

The main change is a substantial increase in intensity within the low-frequency region. This increase in intensity is apparently due to the increase in the intensity of the Boson peak, a feature characteristic of the amorphous state [39]. It was suggested in [40] that, upon introducing REIs into glasses, the Boson peak is influenced by the average distance between REIs surrounded by non-bridging anions—in this case, non-bridging sulfur. Note that the introduction of 0.9 at.% Pr in glasses containing 10 mol.% LaS_1.5_ leads to a significant increase in the scattering intensity in the low-frequency region (Figure 5B,C). This effect is much more pronounced compared with the introduction of La into the original glass (Figure 4A). This may indicate that the positions occupied by La^3+^ and Pr^3+^ ions in the glass structure are non-equivalent. The introduction of Pr^3+^ ions appears to lead to a more substantial formation of non-bridging sulfur.

#### 3.2.3. Peculiarities of the Raman Spectra in the High-Frequency Region

**Figure 6:**
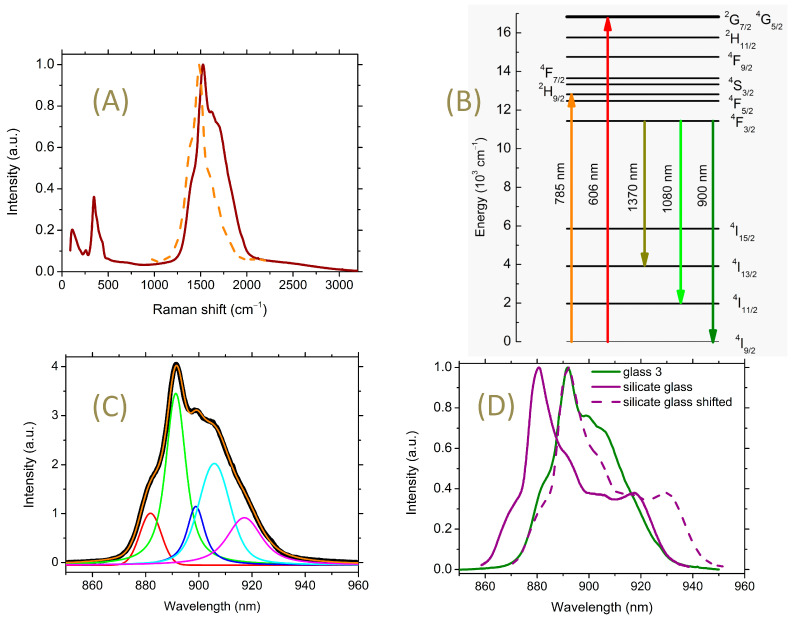
(**A**) The solid brown line is Raman spectrum of glass composition 3; the dashed orange line is absorption spectrum of glass composition 36.7GaS_1.5_-63.3GeS_2_ containing 0.1 at.% Nd recalculated for the dependence on Raman shift. (**B**) Diagram of Nd^3+^ ion levels. The arrows indicate the pump and observed luminescence wavelengths used in this work. (**C**) Luminescence spectrum of Nd^3+^ in glass composition 3 obtained from a part of the Raman spectrum (**A**) and the result of its approximation by 5 peaks. (**D**) Solid lines are Nd^3+^ luminescence spectra corresponding to the ^4^F_3/2_-^4^I_9/2_ transition in silicate glass [42] and glass of composition 3. The dotted line indicates the spectrum of silicate glass shifted along the abscissa axis by 11 nm to the long-wavelength region of the spectrum.

When measuring the Raman spectrum of glasses containing La_2_S_3_ across a wide spectral range, an exceptionally strong signal is observed in the region from 1250 to 2000 cm^−1^ in addition to the Raman spectrum itself (Figure 6A). This signal cannot be explained through the Raman effect.

This phenomenon can be explained by the challenge in separating rare earth elements from one another. For instance, chemically pure Pr (PN 263176 Sigma-Aldrich, Saint Louis, MO, USA) contains 4143.8 ppm Nd. Similarly, chemically pure La contains traces of Pr and Nd. Additionally, the use of 785 nm for recording Raman spectra can lead to the excitation of Nd^3+^ ions to the ^2^H_9/2_ level. This is followed by the radiative transition ^4^F_3/2_-^4^I_9/2_ with a wavelength in the region of 900 nm (Figure 6B), which corresponds to the observed Raman shift of 1627 cm^−1^ at an excitation wavelength of 785 nm.

Furthermore, if we compare the spectral position and shape of the observed signal and the absorption spectrum of Nd^3+^ ions in glass (Figure 6A), we see that they are very similar. Hence, it can be argued that the mentioned feature in the Raman spectra is due to the luminescence of impurity Nd^3+^ ions. Subsequently, these spectra will be referred to as luminescence spectra.

It is noteworthy that these luminescence spectra show Stark splitting. The Coulomb field and the overlap of 4f electrons of the REI shell with the ligand shells result in the splitting of the REI levels in glasses as well as in crystals. In glasses, due to the disordered structure, the transitions between sublevels are usually broadened, which leads to their overlap and smearing. This broadening complicates the isolation of individual transitions between sublevels.

The ^4^F_3/2_-^4^I_9/2_ transition cannot be attributed to hypersensitive transitions, for which the condition ΔS = 0, ΔL ≤ 2, and ΔJ ≤ 2 must be satisfied [41]. However, in the case under study, nevertheless, a certain fine structure is observed. Considering that the Stark splitting is associated with the features of the REI structural surrounding, this allows for some assumptions about the features of this environment.

According to published data, the Nd^3+ 4^F_3/2_ level in silicate glasses splits into two sublevels, while the ^4^I_9/2_ ground state splits into five sublevels [42,43]. Thus, the ^4^F_3/2_-^4^I_9/2_ transition could potentially consist of 10 sub-transitions. For the studied glasses, the corresponding luminescence spectrum is well approximated by at least five peaks (Figure 6C). Assuming that the inhomogeneous broadening for all sub-transitions should be approximately the same in magnitude, we should anticipate at least eight transitions.

Figure 6D shows the Nd^3+^ luminescence spectrum corresponding to this transition in silicate glass [42] and glass of composition 3. The character of the spectra for both glasses is similar in the high-frequency region. This is especially evident when the peak maxima coincide due to the spectral shift of one spectrum relative to the other by 11 nm along the abscissa axis (Figure 6D). The main difference lies in the absence of luminescence for the chalcogenide glass within the spectral range of ~930–940 nm. This range corresponds to sub-transitions with the lowest energy. Apparently, transitions with the lowest energy from the lower ^4^F_3/2_ sublevel to the upper ^4^I_9/2_ sublevels are less typical for chalcogenide glass. This difference could arise from the features of the environment of the Nd^3+^ ion in chalcogenide glass as well as potential channels of nonradiative relaxation. For example, the energy of a possible cross-relaxation transition ^4^I_13/2_-^4^F_9/2_ is approximately around 10,800 cm^−1^.

The similarity of the luminescence spectra may indicate the closeness of the coordination values of the Nd^3+^ ion in the silicate and chalcogenide glasses. Various sources suggest that REIs in silicate glasses have coordination numbers ranging from 6 to 8 [43,44,45]. Concerning chalcogenide glasses, a study of the (La_2_S_3_)_0.07_(Ga_2_S_3_)_0.33_(GeS_2_)_0.60_ system via X-ray and neutron diffraction led to the assumption that La has a coordination of 8 [46]. However, according to another source, rare earth elements in chalcogenide glasses realize a coordination of 6 [7]. Thus, for chalcogenide glasses we have the same interval of uncertainty of the REI coordination value from 6 to 8, although formally, according to Polling, the coordination of REI in oxide glass should be higher than in sulfide glass (see Supplementary Materials).

Such a large shift in the spectral position of the ^4^F_3/2_-^4^I_9/2_ Nd^3+^ luminescent transition in chalcogenide glass compared with silicate (11 nm) is due to the large nephelauxetic effect. This phenomenon means that the increased degree of covalence of the Nd-S bond compared with the Nd-O bond leads to a decrease in the Coulomb interelectron interaction in the 4f shell and thus lowers the energy of the excited levels.

**Figure 7:**
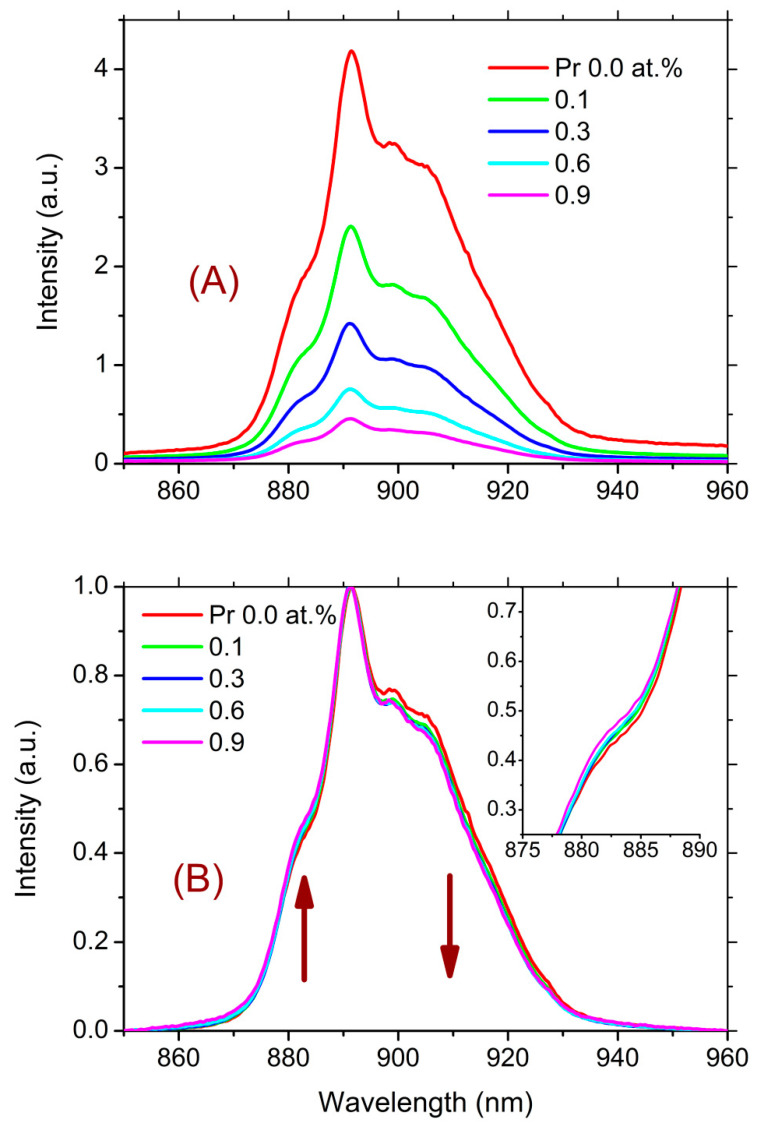
(**A**) Luminescence spectra of Nd^3+^ impurity ions in glass of composition 5 with different Pr content. (**B**) Luminescence spectra of Nd^3+^ impurity ions in glass of composition 5 at different Pr contents normalized to the peak intensity in the region of 891 nm. The arrows indicate the direction of intensity change with increasing Pr content. The inset shows the region of the spectrum on an enlarged scale.

When fractions of a percentage of Pr are introduced into the studied glasses, the luminescence intensity of the impurity Nd^3+^ ions drops sharply. This can be seen for the glass of composition 5 with the addition of Pr (Figure 7A).

Such a drop can be caused by the hetero-ionic cross-relaxation process ^4^F_3/2_-^4^I_9/2_ (Nd^3+^) → ^3^H_6_-^1^D_2_ (Pr^3+^). In addition, it seems that Nd^3+^ and Pr^3+^ ions form joint regions of their increased concentration.

This effect becomes even more pronounced as the relative content of Ga_2_S_3_ decreases in the glass composition. Taking into account that GaS_4/2_^−^ complex structural units contribute to the dissolution of REIs in the chalcogenide glass matrix, it can be concluded that the introduction of Pr increases the degree of inhomogeneity of the REI distribution.

Furthermore, with the introduction of Pr, there is a barely noticeable trend toward a reduction in the relative intensity of low-energy sub-transitions and an increase in higher-energy sub-transitions (as shown in Figure 7B). This trend may indicate a change in the structural environment of Nd^3+^ ions, apparently due to the appearance of Pr^3+^ ions in the second or third coordination spheres. This interpretation aligns with the decrease in the luminescence intensity due to concentration quenching (see Figure 7A).

### 3.3. Luminescent Properties

**Figure 8:**
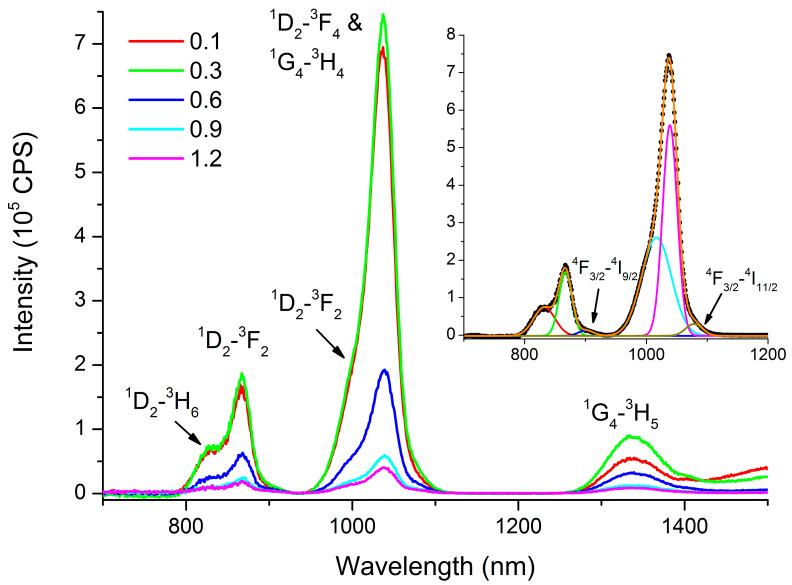
Luminescence spectrum of glass of composition 1 containing 0.1, 0.3, 0.6, 0.9, and 1.2 at.% Pr. The inset shows the result of spectrum fitting for the glass containing 0.3 at.% Pr. Dots are the experimental data.

For glass of composition 1, the maximum luminescence intensity is observed for a composition containing 0.3 at.% Pr under the used excitation and measuring conditions (Figure 8).

As concentrations increase beyond this threshold, luminescence intensity diminishes due to concentration quenching. At Pr concentrations above 0.3 at.%, the interaction between Pr^3+^ ions interconnected via sulfur becomes more prominent, facilitating energy exchange.

**Figure 9:**
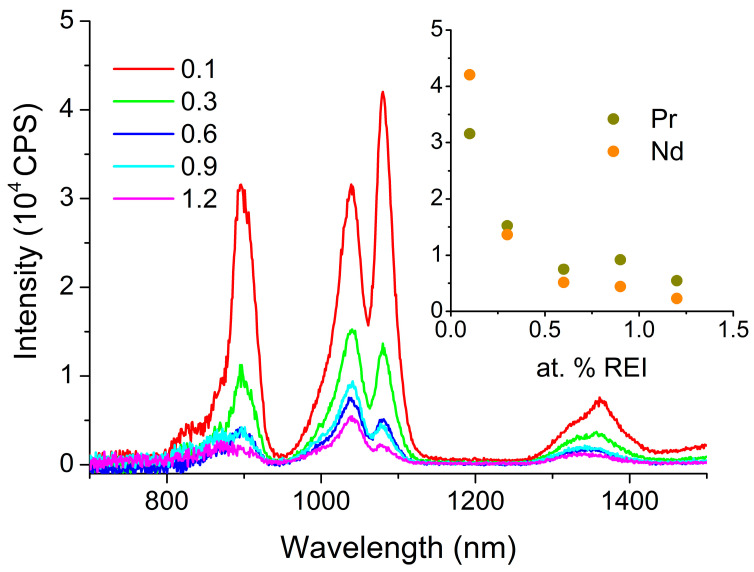
Luminescence spectrum of glass of composition 3 containing 0.1, 0.3, 0.6, 0.9, and 1.2 at.% Pr. The inset shows the dependence of the peak intensity at 1040 nm (Pr^3+^) and 1080 nm (Nd^3+^) on the added Pr concentration.

It is noteworthy that, in addition to the luminescence bands of the Pr^3+^ ions, there are luminescence bands of Nd^3+^ ions corresponding to the ^4^F_3/2_-^4^I_3/2_ and ^4^F_3/2_-^4^I_9/2_ transitions. This can be seen from the result of fitting the luminescence spectrum shown in the inset of Figure 8. First, as indicated in Section 3.2.3, it is challenging to completely separate RE elements from each other. Pure Pr and La contain significant traces of Nd. Second, upon excitation by radiation with a wavelength of 607 nm, both the transition of Pr^3+^ ions to the excited level ^1^D_2_ and the transition of Nd^3+^ ions to the excited levels ^2^G_7/2_ and ^4^G_5/2_ occur. This is especially evident when La (10 mol.% LaS_1.5_) is added to the glass composition, which, as noted above, initially contains an Nd impurity (Figure 9).

From the data presented in Figure 9, three key observations emerge. Firstly, concentration quenching is observed even at the minimum studied concentration, 0.1 at.% Pr. Secondly, at a Pr concentration of 0.1 at.%, the luminescence intensity of Nd^3+^ ions surpasses that of Pr^3+^ ions, despite the difference in concentrations. Thirdly, the luminescence intensity of Nd^3+^ ions decreases with increasing concentrations of Pr^3+^ ions faster than the luminescence intensity of the Pr^3+^ ions themselves.

Despite the fact that La and Pr are neighbors in the lanthanide group, the addition of La to the glass composition not only does not reduce the concentration quenching, but even increases it. This may indicate that Pr and Nd do not isomorphically replace La in the glass network. Instead, regions enriched with both Pr and Nd are formed. This leads to energy exchange between the Pr and Nd ions, among other effects.

The significantly higher luminescence intensity of Nd ions compared with Pr ions, despite their disparate concentrations, can be due to two reasons. First, when a Nd ion is excited to the ^2^G_7/2_ and ^4^G_5/2_ double levels, it rapidly undergoes nonradiative relaxation through a series of closely spaced levels to the ^4^F_3/2_ luminescent level. This ^4^F_3/2_ level possesses a considerably lower energy than the tails of the glassy matrix band stemming from defect states. Conversely, in the case of Pr, the ^1^D_2_ level is separated from the underlying level by a significant energy gap. As a result, ions may relinquish energy to the glass network because this energy level resides at the band’s edge in the region of weak absorption (see Figure 1). Secondly, due to the use of pump radiation with a wavelength of 607 nm, which is at least partially absorbed by the glassy matrix, local heating is induced. Consequently, an elevation in temperature can lead to an increase in luminescence quenching for Pr, while this effect is not observed for Nd [47].

The faster drop in the Nd luminescence intensity compared with that of Pr with increasing Pr concentration (as shown in Figure 9) can apparently be due to the fact that the energy of the ^4^F_3/2_ luminescent level of the Nd ion is slightly higher than that of the ^1^G_4_ Pr luminescent level. With increasing Pr concentrations, Nd ions can nonradiatively transfer energy to Pr ions, inducing an inversion of their relative luminescence intensities.

An increase in the relative content of Ga at the same content of La (glass composition 5) does not change the nature of the dependences presented in Figure 9 (see Figure S2).

### 3.4. Parameters of the Judd–Ofelt Theory for Synthesized Glasses

For the Pr^3+^ ion, the 4f^N−1^5d band is low in energy and close to the ^3^P_2_, ^3^P_1_, ^1^I_6_, and ^3^P_0_ levels [48]. It contradicts the assumptions in the Judd–Ofelt theory, according to which the 4f^N−1^5d configuration should be degenerate in energy and have a significant difference in energy from the 4f^N^ configuration [49,50]. To reconcile this discrepancy, numerous alternative approaches have been suggested [51,52,53]. Nevertheless, if we consider levels with energies lower than that of the ^3^P_0_ level (even lower than ^1^D_2_), the standard method remains adequately accurate for the precise prediction of Judd–Ofelt parameters [54]. Moreover, it is worth noting that all observed and utilized absorption bands stem from electronic transitions [55], rendering the magnetic dipole component negligible.

For the calculation, the measured values of density and refractive index (see Table S1) and data from the optical absorption spectra (spectral position of optical transitions and their corresponding integrated cross-sections of optical absorption) were used. The matrix elements for the calculations were referenced from [22]. The calculation results are shown in Table 2 and Table 3. The resulting branching ratios are presented in Table S2. The root-mean-square deviation (RMS) between the theoretical and experimental values of the line strengths was about 0.23 × 10^−20^ cm^2^. It indicates a good level of calculation accuracy.

The obtained values of the parameters Ω_2_, Ω_4_, and Ω_6_ are consistent with the reference data for the Ge_25_Ga_5_S_70_ composition (Ω_2_ = 12.8, Ω_4_ = 4.3, Ω_6_ = 7.7) [56].

The value of the parameter Ω_2_ is related to the degree of covalence of the REI bonds and the symmetry of its environment [55,57,58]. According to the acquired data, the addition of La_2_S_3_ to the glasses results in a reduction in covalence (or increased symmetry). Alterations in the Ga_2_S_3_ content relative to GeS_2_, while maintaining a constant La_2_S_3_ content, display minimal influence on the Ω_i_ parameters (where I = 2, 4, 6). The highest calculated radiation probability is observed for the ^1^G_4_-^3^H_5_ transition, corresponding to a wavelength of 1.336 μm (one of the wavelengths of communication lines). Among the studied glass compositions, the highest radiation probability and the lowest radiation lifetime are observed for the composition with the highest content of La_2_S_3_ and Ga_2_S_3_ (composition 5). The increase in emission probability with increasing La content in the glasses is mainly due to the increase in the refractive index (see Table S1). This observation aligns with the correlation between the Ω_4_/Ω_6_ ratio and stimulated emissivity [58]. In the case of composition 5, the Ω_4_/Ω_6_ ratio attains its maximum value.

## 4. Conclusions

The incorporation of La_2_S_3_ into Ga_2_S_3_-GeS_2_ glasses leads to structural changes, including a reduction in the proportion of Ga-Ga bonds (resulting in an increase in the relative content of GaS_4/2_ tetrahedra), an increase in three-coordinated sulfur and non-bridging sulfur, and a decrease in the fraction of GeS_4/2_ tetrahedra linked by edges.

The introduction of Pr into glasses leads to a greater increase in the concentration of non-bridging sulfur compared with the addition of La. This difference suggests that the places occupied by La^3+^ and Pr^3+^ ions in the glass structure are not equivalent.

Optical absorption spectroscopy data and Judd–Ofelt theory calculations indicate that the ionicity of the Pr^3+^ ions’ bonds increases upon the addition of La_2_S_3_ to the glasses.

When studying the structure and luminescent properties of La-containing glasses, it is necessary to take into account a significant concentration of traces of RE elements (Pr and Nd). At low Pr concentrations (≤0.1 at.%), the luminescence intensity of impurity Nd^3+^ ions upon excitation at a wavelength of 607 nm is even higher than that of the added Pr^3+^ ions despite the difference in concentrations. This phenomenon is a result of the energy level distribution of REIs and the spectral position of the fundamental absorption edge of glass. However, when fractions of a percent of Pr are introduced into the studied glasses, the luminescence intensity of impurity Nd^3+^ ions drops sharply. This effect can be caused by the hetero-ionic cross-relaxation process ^4^F_3/2_-^4^I_9/2_ (Nd^3+^) → ^3^H_6_-^1^D_2_ (Pr^3+^). In addition, the Nd^3+^ and Pr^3+^ ions apparently form joint regions of their increased concentration in the glass.

Despite the fact that La_2_S_3_ with Ga_2_S_3_ and GeS_2_ forms a large region of glass formation, La_2_S_3_ does not contribute to a decrease in the concentration quenching of the luminescence of the Pr^3+^ ions in these glasses. At the same time, if concentration quenching is not taken into account, then, according to Judd–Ofelt theory calculations, among the studied glass compositions, the highest radiation probability and the shortest radiative lifetime are observed for the composition with the highest content of La_2_S_3_ and Ga_2_S_3_.

Without taking into account concentration quenching, calculations according to the Judd–Ofelt theory show that among the studied glass compositions, the highest radiation probability and the shortest radiation lifetime are observed for the composition with the highest content of La_2_S_3_ and Ga_2_S_3_.

Thus, when developing luminescent materials based on chalcogenide glasses containing La, the following points must be taken into account. Despite the widely cited opinion that the presence of La in glasses promotes the dissolution of other RE elements in them, our findings suggest otherwise. La does not reduce the heterogeneity of the distribution of RE elements in the glass matrix and therefore does not increase the threshold for concentration quenching of luminescence. However, its presence in the glass enhances luminescence intensity. Additionally, the introduction of significant amounts of La introduces traces of Pr and Nd, the presence of which must be taken into account when studying and interpreting luminescent properties.

## Figures and Tables

**Table 1:** Compositions of the synthesized glassy matrixes.

No Composition	GaS_1.5_ (mol.%)	GeS_2_ (mol.%)	LaS_1.5_ (mol.%)
1	25.00	75.00	0.00
2	24.25	72.75	3.00
3	22.50	67.50	10.00
4	47.00	53.00	0.00
5	42.30	47.70	10.00

**Table 2:** Judd–Ofelt parameters for glasses of compositions 1–5 with Pr^3+^ ions.

No Composition	Ω_2_ (10^−20^ cm^2^)	Ω_4_ (10^−20^ cm^2^)	Ω_6_ (10^−20^ cm^2^)	Radiative Lifetime from ^1^G_4_, ms
1	14.28	5.19	7.23	0.411
3	10.68	6.28	6.43	0.366
5	10.53	6.10	6.00	0.334

**Table 3:** Calculated transition probabilities in the studied glasses. Numbers 1–5 indicate the number of composition.

Transition	Transition Probabilities, s^−1^		
	1	3	5
^1^G_4_-^3^F_4_	88.9	101.1	108.5
^1^G_4_-^3^F_3_	13.8	15.4	16.6
^1^G_4_-^3^F_2_	10.5	14.3	15.8
^1^G_4_-^3^H_6_	798.7	855.4	960.5
^1^G_4_-^3^H_5_	1356.0	1554.1	1685.4
^1^G_4_-^3^H_4_	166.0	193.8	209.8

## Data Availability

Data are contained within the article.

## Supplementary Materials

The following supporting information can be downloaded at: https://www.mdpi.com/article/10.3390/ma16227094/s1.
