# Supplementary material for: Peculiarity of the Structure and Luminescence of Glasses in La2S3-Ga2S3-GeS2:Pr3+ System
Source: Materials (Basel). 2023 Nov 9;16(22):7094. doi: 10.3390/ma16227094 (PMC10672385; doi:10.3390/ma16227094)
Supplement: Supplementary file 1 [file materials-16-07094-s001.zip › materials-2609078-supplementary.pdf]

Supplementary

1. Ampoule graphitization method.

The graphitization method is based on the reaction of thermal decomposition of acetone vapors on the heated walls of the ampoule. By varying the rate of supply of acetone vapor, the temperature of the walls of the ampoule, and the reaction time, it becomes possible to obtain a glassy carbon layer of a specified thickness that adheres strongly to the quartz walls of the ampoule.

2. The examples of X-ray diffraction pattern and photo image of the synthesized glasses.

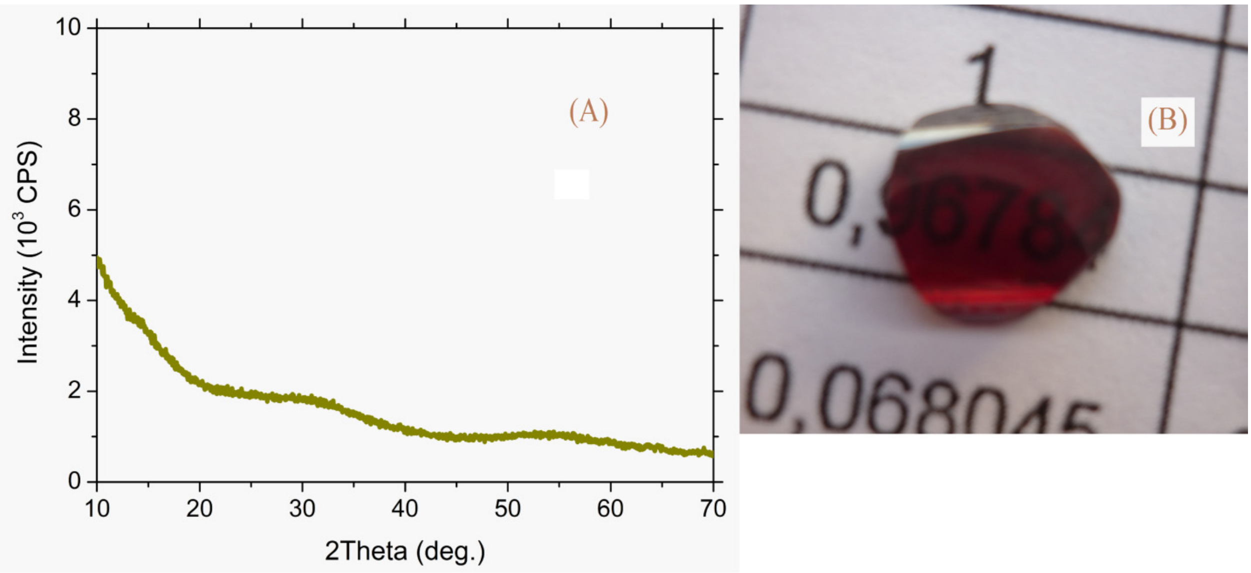

Figure S1. (A) X-ray diffraction pattern of the glass with composition 3 with 0.6 at. % Pr. (**B**) Photo image of the glass with composition 5 with 0.3 at. % Pr placed on paper with the printed text.

3. Coordination of REI.

Formally, according to Polling's theory, the coordination is determined by the ratio of the ionic radii between the cation and anion. With a ratio falling within the range of 0.414-0.732, the REI should occupy the 6th coordinate position. For a ratio in the range of 0.732-0.904, the REI can exhibit coordination of up to 9 [12]. Taking into account the ionic radii of La^{3+} (1.03 Å), O^{2-} (1.36 Å), and S^{2-} (1.82 Å), it can deduced than the coordination number of La in sulfide glass should be 6, while in oxide glass, it should exceed 6.

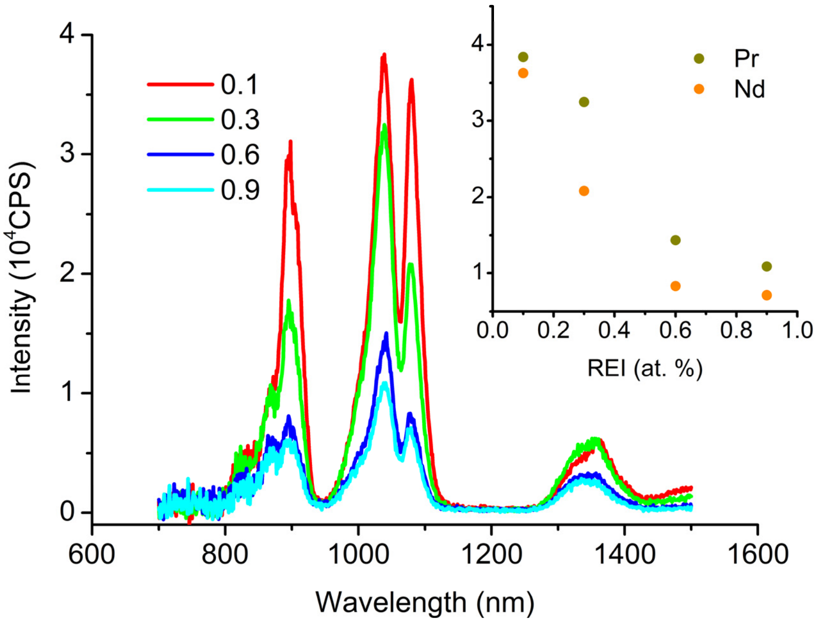

Figure S2. The luminescence spectrum of glass composition 5 containing 0.1, 0.3, 0.6, 0.9 at. % Pr. The inset shows the dependence of the peak intensity at 1040 nm (Pr^{3+}) and 1080 nm (Nd^{3+}) on the concentration of added Pr.

Table S1. The measured values of density and refractive index of the studied glass hosts.

Glass composition	Density (g/cm3)	Refractive index
1	2,95	2,08
2	2,98	2,12
3	3,14	2,21
5	3,33	2,29

Table S2. The calculated values of the branching ratio in the studied glasses.

transition	Branching ratio		
	Glass 1	Glass 3	Glass 5
$^1G_4$-$^3F_4$	0.036	0.037	0.036
$^1G_4$-$^3F_3$	0.006	0.006	0.006
$^1G_4$-$^3F_2$	0.004	0.005	0.005
$^1G_4$-$^3H_6$	0.328	0.313	0.321
$^1G_4$-$^3H_5$	0.557	0.568	0.562
$^1G_4$-$^3H_4$	0.068	0.071	0.070
